# Risk factors to bloodstream infection due multidrug-resistant Results *Acinetobacter baumanni *in colonized patients in the ICU

**DOI:** 10.1186/cc9646

**Published:** 2011-03-11

**Authors:** R Passos, S Ultchak, P Mota, AV Mendes, M Souza, RH Oliveira, PB Batista

**Affiliations:** 1 Sao Rafael, Sao Rafael, Brazil

## Introduction

Epidemic outbreaks caused by multidrug-resistant *Acinetobacter *spp. (MDR Aspp) in ICUs have emerged in recent years. The incidence of MDR Aspp bacteremia, which develops as a result of colonization, is increasing through widespread dissemination of the pathogen and may cause severe clinical disease that is associated with a high mortality. The aim of the study was to evaluate risk factors for MDR Aspp bacteremia in patients colonized with MDR Aspp in the ICU.

## Methods

We conducted a prospective, observational study of all patients colonized with MDR Aspp in the ICU between January 2007 and December 2010. Screening for MDR Aspp (using axillary, oropharynx and rectal swabs) was performed weekly. Only the first bacteremia was considered.

## Results

Of the 185 patients colonized with MDR AB, 74 developed MDR Aspp bacteremia. APACHE II and SOFA scores were higher in bacteremic than nonbacteremic patients at the time of ICU admission (22 vs. 16; *P *= 0.015, 16 vs. 9; *P *< 0.001, respectively). There was no difference between the two groups in the duration of time from ICU admission to colonization (8.2 vs. 7.8 days; *P *= 0.923). In univariate analysis, advanced age, admission for clinical reason, use of broad-spectrum antibiotic agents, total parenteral nutrition, having a central venous catheter, endotracheal tube, arterial catheter or nasoenteral tube and acute renal failure requiring dialysis were significant risk factors for bacteremia (all *P *< 0.05). In multivariate analysis, the number of recent invasive procedures (OR, 4.17; 95% CI, 1.6 to 11.1; *P *= 0.001) and previous administration of carbapenem (OR, 2.07; 95% CI, 1.47 to 2.91; *P *= 0.036) were independently associated with MDR Aspp bacteremia.

## Conclusions

Our results suggest that the nosocomial occurrence of MDRA spp bacteremia in colonized patients is strongly related to the number of invasive procedures and may be favored by the selection pressure of previous carbapenem administration.

